# Exploring Oxidative Stress Mechanisms of Nanoparticles Using Zebrafish (*Danio rerio*): Toxicological and Pharmaceutical Insights

**DOI:** 10.3390/antiox14040489

**Published:** 2025-04-18

**Authors:** Denisa Batir-Marin, Monica Boev, Oana Cioanca, Ionut-Iulian Lungu, George-Alexandru Marin, Ana Flavia Burlec, Andreea-Maria Mitran, Cornelia Mircea, Monica Hancianu

**Affiliations:** 1 Department of Pharmaceutical Sciences, Faculty of Medicine and Pharmacy, “Dunărea de Jos” University, 800008 Galati, Romania; denisa.batir@ugal.ro; 2 Faculty of Pharmacy, “Grigore T. Popa” University of Medicine and Pharmacy, 16 University Street, 700115 Iasi, Romania; ionut-iulian.lungu@umfiasi.ro (I.-I.L.); george17marin@gmail.com (G.-A.M.); ana-flavia.l.burlec@umfiasi.ro (A.F.B.); andreea-maria.mitran@umfiasi.ro (A.-M.M.); cornelia.mircea@umfiasi.ro (C.M.); monica.hancianu@umfiasi.ro (M.H.)

**Keywords:** nanoparticles, oxidative stress, zebrafish, pharmaceutical applications

## Abstract

Nanoparticles (NPs) have revolutionized biomedical and pharmaceutical applications due to their unique physicochemical properties. However, their widespread use has raised concerns regarding their potential toxicity, particularly mediated by oxidative stress mechanisms. This redox imbalance, primarily driven by the overproduction of reactive oxygen species (ROS), plays a central role in NP-induced toxicity, leading to cellular dysfunction, inflammation, apoptosis, and genotoxicity. Zebrafish (*Danio rerio*) have emerged as a powerful in vivo model for nanotoxicology, offering advantages such as genetic similarity to humans, rapid development, and optical transparency, allowing real-time monitoring of oxidative damage. This review synthesizes current findings on NP-induced oxidative stress in zebrafish, highlighting key toxicity mechanisms and case studies involving metallic (gold, silver, copper), metal oxide (zinc oxide, titanium dioxide, iron oxide), polymeric, and lipid-based NPs. The influence of NP physicochemical properties, such as size, surface charge, and functionalization, on oxidative stress responses is explored. Additionally, experimental approaches used to assess ROS generation, antioxidant enzyme activity, and oxidative damage biomarkers in zebrafish models are examined. In addition to toxicity concerns, pharmaceutical applications of antioxidant-modified NPs are evaluated, particularly their potential in drug delivery, neuroprotection, and disease therapeutics. Notably, studies show that curcumin- and quercetin-loaded nanoparticles enhance antioxidant defense and reduce neurotoxicity in zebrafish models, demonstrating their promise in neuroprotective therapies. Furthermore, cerium oxide nanoparticles, which mimic catalase and SOD enzymatic activity, have shown significant efficacy in reducing ROS and protecting against oxidative damage. Challenges in zebrafish-based nanotoxicology, the need for standardized methodologies, and future directions for optimizing NP design to minimize oxidative stress-related risks are also discussed. By integrating insights from toxicity mechanisms, case studies, and pharmaceutical strategies, this review supports the development of safer and more effective nanoparticle-based therapies while addressing the challenges of oxidative stress-related toxicity.

## 1. Introduction

Nanoparticles (NPs) are increasingly utilized across industry in pharmaceuticals, biomedical imaging, drug delivery, cosmetics, diagnostics, and environment-related products and services [1]. Due to their small size and high surface area-to-volume ratio, NPs exhibit unique physicochemical properties that enhance their reactivity and bioavailability, making them ideal for various biomedical and technological applications [2]. In the pharmaceutical field, NPs serve as efficient drug carriers, improving drug solubility, targeted delivery, and controlled release, reducing side effects and enhancing therapeutic efficacy [3]. Metallic NPs, such as gold (Au) and silver (Ag), are extensively applied in biomedical imaging and diagnostics due to their optical and electronic properties [4]. Additionally, lipid-based and polymeric NPs are commonly employed in drug delivery systems, offering enhanced biocompatibility and reduced toxicity [5]. Beyond biomedical applications, NPs are widely used in cosmetics and skincare products, where they enhance product stability and skin penetration [6]. Titanium dioxide (TiO_2_) and zinc oxide (ZnO) NPs, for example, are key ingredients in sunscreens, providing superior UV protection [7]. However, concerns regarding the potential toxicity of NPs, especially their ability to generate oxidative stress, have increased [8]. Environmental exposure to NPs through industrial waste, air pollution, and contaminated water sources has raised significant health concerns, necessitating further research into their long-term effects on biological systems [9].

One of the most critical toxicological effects associated with NPs is oxidative stress, which occurs when NPs induce an imbalance between the production of reactive oxygen species (ROS) and the antioxidant defense mechanisms within biological systems [10]. ROS are highly reactive molecules—this includes moieties such as the superoxide anion (O_2_^−^), hydrogen peroxide (H_2_O_2_), and hydroxyl radicals (OH^−^)—and play a crucial role in cellular signaling, but have the potential of being harmful when generated excessively [11]. Overproduction of ROS leads to oxidative damage to lipids, proteins, and nucleic acids, contributing to cellular dysfunction and various pathological conditions [12].

Numerous studies have demonstrated that exposure to metal and metal oxide NPs, such as AgNPs, CuO NPs, and ZnO NPs, results in increased ROS production, leading to oxidative stress-induced apoptosis and genotoxicity [13]. The oxidative stress triggered by NPs can activate multiple pathways, including mitochondrial dysfunction, DNA damage, and inflammatory responses [14]. Prolonged oxidative stress has been linked to chronic inflammation, which plays a crucial role in the pathogenesis of various diseases, including cancer, neurodegeneration, and cardiovascular disorders [15]. Understanding oxidative stress as a central mechanism of NP toxicity is essential for developing safer nanomaterials with reduced adverse effects.

Zebrafish (*Danio rerio*) have emerged as a widely used vertebrate model in nanotoxicology due to their genetic similarity to humans (~70%), rapid development, and ease of maintenance in laboratory settings [16]. One of the key advantages of zebrafish is the optical transparency of embryos, allowing real-time visualization of NP interactions and oxidative stress responses in developing tissues [17]. This feature makes zebrafish an excellent in vivo model for assessing NP-induced oxidative damage, mitochondrial dysfunction, and inflammatory responses [15].

**Figure 1:**
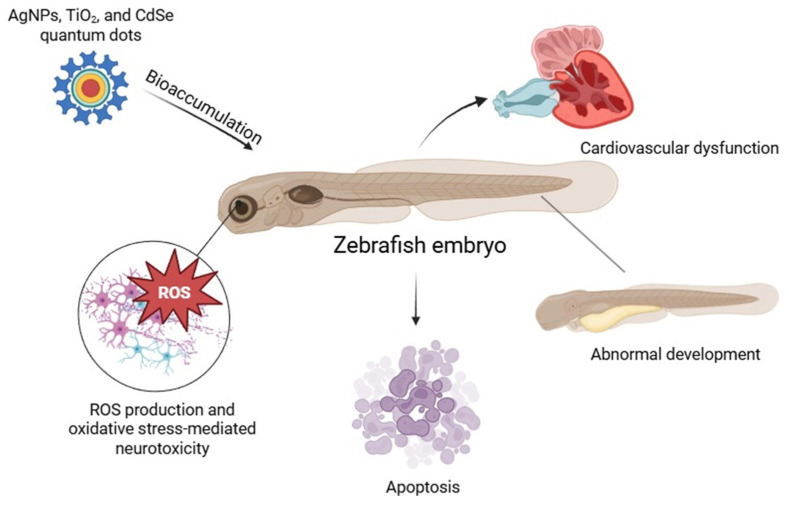
Nanoparticle-induced oxidative stress and developmental toxicity in zebrafish embryos.

Studies have shown that zebrafish embryos exposed to AgNPs, TiO_2_ NPs, and CdSe quantum dots exhibit significant generation of ROS, leading to apoptosis and developmental abnormalities [18]. In particular, AgNPs have been reported to induce oxidative stress-mediated neurotoxicity and cardiovascular dysfunction in zebrafish models [12]. The use of zebrafish in NP toxicity assessments provides valuable insights into the biodistribution of NP, bioaccumulation, and their effects on different organ systems. Furthermore, zebrafish models are increasingly employed in high-throughput screening assays to evaluate pharmaceutical formulations and antioxidant-modified NPs designed to mitigate oxidative stress (Figure 1) [19].

This review is a comprehensive analysis of research on NP-induced oxidative stress in zebrafish, focusing on the implications in toxicology and pharmaceutical applications. Key mechanisms of oxidative stress are highlighted to evaluate case studies which involve different types of NPs, with zebrafish as a model species with therapeutic potential. Additionally, the review addresses challenges in the field of nanotoxicology and the need for standardized methodologies to improve risk assessment for NPs and pharmaceutical development.

## 2. Physicochemical Properties of Nanoparticles Influencing Oxidative Stress

### 2.1. NP Size and Surface Area

The size of nanoparticles plays a crucial role in their toxicity potential, as smaller NPs exhibit higher surface-area-to-volume ratios, increasing their reactivity and potential to induce oxidative stress [20]. Studies have demonstrated that reduced NP size correlates with increased ROS generation and toxicity in aquatic organisms, including zebrafish. For instance, silver nanoparticles (AgNPs) smaller than 10 nm have been shown to trigger higher oxidative stress levels compared to larger particles due to their enhanced cellular penetration and bioavailability [21,22]. Similarly, titanium dioxide (TiO_2_) NPs of <20 nm have been reported to induce significant ROS production and mitochondrial dysfunction in zebrafish embryos [23,24]. Table 1 presents a synthesis of studies on the size of various NPs and their induced oxidative stress.

Silver Nanoparticles (Ag NPs): Zebrafish embryos exposed to AgNPs of varying sizes demonstrated that smaller AgNPs (10 nm) triggered more severe ROS production and developmental toxicity compared to larger particles (50 nm) [21]. The study indicated that smaller AgNPs disrupted the antioxidant defense system, downregulating antioxidant enzymes such as superoxide dismutase (SOD) and catalase (CAT) [44].

Gold Nanoparticles (Au NPs): Investigations on AuNPs found that particles smaller than 20 nm induced more oxidative stress than larger ones due to their ability to accumulate in tissues and penetrate cellular compartments [45]. Additionally, smaller AuNPs enhanced pro-inflammatory cytokine expression, further exacerbating ROS generation [46,47].

Copper Nanoparticles (Cu NPs) have gained attention in nanotoxicology due to their unique physicochemical properties and potential biomedical applications [36]. Their small size (≤20 nm) leads to increased oxidative stress, causing significant DNA damage and mitochondrial dysfunction in biological systems [48]. Studies have demonstrated that Cu NPs induce oxidative stress through ROS overproduction, which can trigger inflammation and apoptosis in zebrafish tissues [35,49].

Iron Nanoparticles (Fe NPs) are widely used in environmental remediation and biomedical applications. Their unique magnetic properties make them valuable for drug delivery, hyperthermia treatment, and MRI contrast agents [39]. Studies show that Fe NPs generate ROS through Fenton reactions, causing cellular damage and mitochondrial dysfunction in zebrafish models [50]. Exposure to Fe NPs in zebrafish embryos has been associated with oxidative stress-induced apoptosis, DNA damage, and developmental abnormalities [51].

Zinc Oxide Nanoparticles: Different sizes were evaluated for oxidative stress induction in zebrafish larvae, revealing that smaller ZnO NPs led to significantly higher ROS production and mitochondrial damage [31,33]. The study suggested that the smaller NPs had increased bioavailability, leading to prolonged oxidative stress and apoptosis [34,52].

Titanium Dioxide Nanoparticles (TiO_2_ NPs): A recent study demonstrated that TiO_2_ NPs induce oxidative stress and neurotoxic effects in zebrafish embryos, disrupting behavioral responses and mitochondrial function [24]. These findings suggest that TiO_2_ NPs contribute to ROS accumulation and affect neural pathways, highlighting their potential long-term toxicity in aquatic organisms and human health implications [23].

Copper Oxide Nanoparticles (CuO NPs) are widely utilized in industrial and biomedical applications due to their antimicrobial properties and catalytic activity [36]. Studies have shown that CuO NPs induce substantial ROS production, causing lipid peroxidation, mitochondrial dysfunction, and DNA fragmentation in zebrafish models. Exposure to CuO NPs in zebrafish embryos has been linked to apoptotic cell death and developmental toxicity due to their ability to disrupt redox homeostasis [28].

Iron(III) Oxide Nanoparticles (Fe_2_O_3_ NPs) have superparamagnetic properties. Studies have indicated that Fe_2_O_3_ NPs can induce oxidative DNA fragmentation, lipid peroxidation, and mitochondrial dysfunction in zebrafish models, leading to apoptosis and neurotoxicity [53]. Additionally, zebrafish embryos exposed to Fe_2_O_3_ NPs exhibit inflammatory responses and oxidative stress in gill and liver tissues due to ROS-induced damage [50,54].

Cadmium Selenide Quantum Dots (CdSe QDs) have optoelectronic applications due to their fluorescent properties. Studies have demonstrated that CdSe QDs induce DNA fragmentation, lipid peroxidation, and apoptosis in zebrafish models, leading to developmental abnormalities and neurotoxicity [55]. Exposure to CdSe QDs has also been linked to mitochondrial dysfunction, as the nanoparticles interfere with electron transport chain activity, leading to increased ROS generation and cellular damage [56].

Zinc Sulfide Quantum Dots (ZnS QDs) have photonic applications due to their unique optical properties. Studies have shown that ZnS QDs can induce lipid peroxidation, oxidative DNA damage, and apoptosis in zebrafish embryos, particularly under illumination conditions [42,57]. Exposure to ZnS QDs has also been linked to mitochondrial stress and inflammatory responses, contributing to oxidative toxicity [43].

These findings reinforce the idea that NP size plays a crucial role in determining oxidative stress outcomes and toxicity levels. Future research should focus on optimizing NP size to balance efficacy in pharmaceutical applications while minimizing oxidative damage.

### 2.2. Surface Charge and Coating Effects

**Figure 2:**
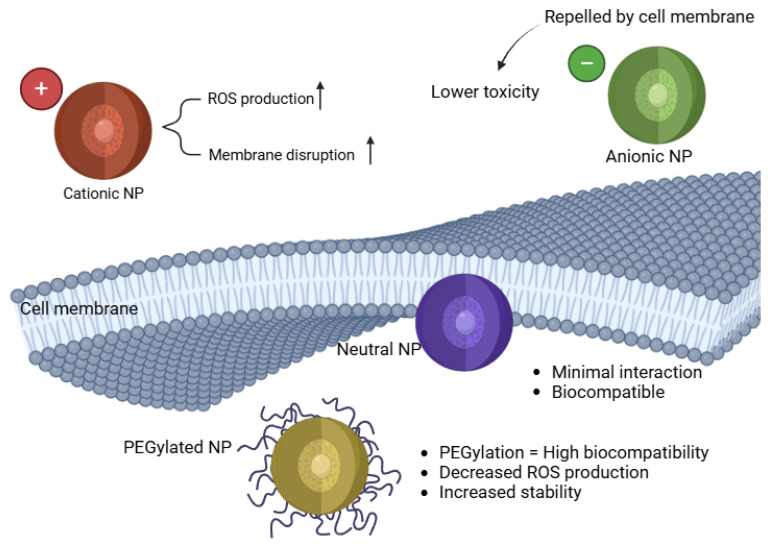
Impact of NP surface charge on cellular uptake and toxicity.

The charge and surface modifications of NPs significantly influence their cellular uptake, bioavailability, and oxidative stress potential. Positively charged (cationic) NPs tend to exhibit greater cytotoxicity due to enhanced cellular uptake and stronger electrostatic interactions with negatively charged cell membranes [58]. Conversely, surface coatings such as polyethylene glycol (PEG) can modify NP interactions and mitigate oxidative stress [59]. Figure 2 represents a schematic of the importance of the surface charge and the coating effects.

In addition to surface charge and chemical coatings, the formation of a biomolecular corona represents a critical factor influencing the biological identity and toxicity of nanoparticles. When NPs enter biological environments, such as zebrafish media or blood plasma, they rapidly adsorb proteins and other biomolecules on their surfaces, forming a dynamic protein corona. This corona can significantly alter nanoparticle properties, including surface charge, hydrodynamic diameter, colloidal stability, and cellular interactions, ultimately affecting biodistribution, oxidative stress responses, and toxicity profiles [60,61]. Recent studies have shown that the corona may either attenuate or exacerbate reactive oxygen species (ROS) production, depending on its protein composition and conformational dynamics [62]. For example, in zebrafish liver cells, protein corona formation was found to influence NP-induced oxidative stress and disrupt glycolipid metabolism [63]. Moreover, time-dependent and species-specific variations in corona composition present challenges for reproducibility and toxicological predictions in vivo [60,61]. Understanding the nanoparticle-protein alliance is thus essential not only for environmental safety assessments, but also for the rational design of nano-therapeutics and precision nanomedicine [61,62].

Cationic NPs show stronger ROS production and cytotoxicity compared to neutral or anionic NPs [64]. These nanoparticles tend to interact more aggressively with negatively charged cell membranes, facilitating their internalization and causing significant oxidative stress. Studies have demonstrated that cationic NPs exhibit increased genotoxicity and inflammatory responses due to their ability to disrupt cell membranes, induce mitochondrial dysfunction, and activate apoptosis pathways [65]. For example, research on AgNPs has shown that positively charged silver nanoparticles are more cytotoxic than their neutral or negatively charged counterparts, leading to increased ROS production and DNA damage in zebrafish embryos [11,66]. Similarly, CuNPs exhibit a strong capacity to generate oxidative stress, leading to lipid peroxidation and cell membrane rupture [67]. Furthermore, cationic NPs have been linked to neuroinflammation and cardiovascular dysfunction in zebrafish models, highlighting the need for careful assessment of surface charge when designing nanoparticle-based applications in biomedical and pharmaceutical sciences [68,69,70].

Anionic and Neutral NPs tend to show reduced oxidative stress responses, as their charge properties limit cellular interactions and membrane permeability, leading to lower toxicity [71]. Studies have previously demonstrated that negatively charged NPs have lower cytotoxicity due to their repulsion from negatively charged cell membranes, reducing internalization and subsequent ROS production. Neutral lipid-based nanoparticles like liposomes have shown that they do not significantly induce ROS generation, making them promising candidates for drug delivery applications [72,73,74]. In another study, negatively charged Au NPs exhibited reduced inflammatory response and minimal oxidative damage compared to positively charged counterparts [75]. The reduced interaction of anionic and neutral NPs with cellular components suggests their potential as safer alternatives in biomedical applications, particularly in drug delivery and antioxidant therapy.

Surface coatings and modifications of NPs play a crucial role in influencing their interactions with biological systems, particularly in mitigating oxidative stress [65]. Functionalizing NPs with polymers such as polyethylene glycol (PEG) or albumin can reduce ROS production by decreasing NP aggregation and limiting cellular internalization. For instance, PEGylation of AuNPs has been shown to diminish oxidative stress and inflammatory responses [76,77]. Similarly, coating AgNPs with biocompatible polymers has been demonstrated to reduce their ROS generation and improve stability in biological environments [78].

As summarized in Table 1, nanoparticle size and surface charge significantly influence oxidative stress outcomes in zebrafish models. Smaller nanoparticles (typically < 50 nm) tend to generate higher levels of ROS due to their larger surface area-to-volume ratio, which enhances cellular uptake and mitochondrial interaction. Positively charged NPs, such as amine-functionalized particles, are more likely to disrupt cellular membranes and increase intracellular ROS, whereas neutral or PEGylated NPs exhibit reduced oxidative potential due to steric stabilization and limited cellular internalization. These trends are visually depicted in Figure 2, which illustrates how surface modifications modulate NP interaction with biological membranes and redox-sensitive organelles. By integrating these physicochemical parameters, researchers can better predict and control the oxidative effects of nanoparticle formulations in vivo.

## 3. The Physicochemical Properties of Nanoparticles Affect Oxidative Stress

NP-induced oxidative stress in zebrafish occurs either through direct ROS generation or secondary mechanisms like inflammation [16]. Excess ROS production disrupts cellular homeostasis, leading to biomolecular damage. One significant effect resulting from exposure to NPs is the inhibition of key antioxidant enzymes, like SOD, CAT, and GPx, weakening the cellular defense system [29]. Exposure to TiO_2_ NPs has been shown to suppress antioxidant defenses in zebrafish liver and gill tissues, resulting in oxidative damage and inflammation [24]. Similarly, AgNPs significantly reduce SOD and CAT activity in zebrafish larvae, increasing oxidative stress and lipid peroxidation [44,79]. CuO NPs further disrupt glutathione homeostasis, impairing detoxification pathways and exacerbating ROS accumulation [80]. Also, ZnO NP exposure depleted antioxidant enzyme levels in zebrafish embryos, correlating with mitochondrial dysfunction and apoptosis [36,81]. These effects are synthetized in Table 2.

### 3.1. Mitochondrial Dysfunction and Apoptotic Pathways

Mitochondria are highly susceptible to oxidative stress caused by NP exposure. ROS accumulation in mitochondria disrupts the function of the electron transport chain (ETC), reduces ATP production, and activates pro-apoptotic signaling pathways [5,36]. NP-induced oxidative damage can trigger p53 tumor suppressor activation, leading to caspase-3-mediated apoptosis. A study on zebrafish embryos exposed to CuO NPs demonstrated significant mitochondrial swelling, cytochrome c release, and p53 activation, which triggers caspase-3-mediated apoptosis. This process has been associated with neurotoxicity in zebrafish brain tissue [86]. Another study found that AgNP exposure led to mitochondrial membrane potential collapse in zebrafish neurons, accelerating neurotoxicity through p53 upregulation and caspase-3 activation [87]. Additionally, TiO_2_ NPs were reported to induce oxidative damage in zebrafish liver cells, impairing mitochondrial respiration and promoting apoptotic cell death [88]. Furthermore, CdSe quantum dots disrupted mitochondrial membrane integrity in zebrafish cardiomyocytes, contributing to cardiac dysfunction and increased apoptotic cell death [89].

### 3.2. Inflammation and Immune Dysregulation

NP exposure in zebrafish has been linked to immune system dysregulation through the activation of pro-inflammatory cytokines such as tumor necrosis factor-alpha (TNF-α), interleukin-6 (IL-6), and nuclear factor-kappa B (NF-κB) [29]. These cytokines are key mediators of inflammation, leading to immune overactivation and subsequent tissue damage. Chronic immune activation contributes to prolonged oxidative stress, affecting multiple organ systems, including the gills, liver, and nervous system [54]. Studies indicate that NP-induced inflammation can also disrupt hematopoiesis and immune cell homeostasis, further exacerbating toxicity. Recent studies have shown that Fe_2_O_3_ NPs not only activate NF-κB pathways in zebrafish gills, leading to oxidative stress and impaired respiratory function, but also increase the expression of IL-8 and IL-1β, leading to macrophage recruitment and localized inflammation [10]. Another study demonstrated that AgNPs cause upregulation of TNF-α and IL-6 in zebrafish liver cells, promoting hepatotoxicity and oxidative damage [90,91]. Additionally, exposure to TiO_2_ NPs in zebrafish larvae resulted in increased leukocyte infiltration and cytokine secretion, demonstrating NP-induced immune activation in early developmental stages [92].

### 3.3. Genotoxicity and Lipid Peroxidation

Oxidative stress induced by NPs can lead to severe genotoxic effects, including DNA fragmentation, chromosomal aberrations, and mutagenic potential, which may contribute to long-term developmental and reproductive toxicity in zebrafish. DNA damage is commonly assessed through comet assays and micronucleus tests, which have revealed increased genotoxic markers upon exposure to metal-based and semiconductor NPs. Additionally, lipid peroxidation, which is caused by the excessive attacks of ROS to membrane lipids, compromises cellular integrity, disrupts signaling pathways, and initiates apoptotic cascades [8]. Studies have shown that oxidative stress-driven lipid peroxidation is a key mechanism in NP-mediated toxicity, particularly in organs with high lipid content such as the brain and liver. CdSe QDs were found to induce DNA strand breaks in zebrafish larvae, leading to chromosomal fragmentation and developmental abnormalities [93]. A study using fluorescent in situ hybridization (FISH) confirmed increased nuclear damage following CdSe QD exposure, indicating their potential to cause long-term genetic instability [94]. Another study demonstrated that exposure to ZnO NPs in zebrafish embryos resulted in both DNA double-strand breaks and increased lipid peroxidation in liver and muscle tissues, further confirming the dual genotoxic and oxidative stress-related impacts of these NPs. Histopathological examination showed mitochondrial swelling and nuclear fragmentation, consistent with apoptotic progression [95].

According to Table 2, different classes of NPs induce oxidative stress via distinct but overlapping mechanisms in zebrafish models. Metal oxide nanoparticles such as TiO_2_ and CuO primarily disrupt mitochondrial function and suppress antioxidant enzymes, leading to apoptosis in liver and brain tissues. Semiconductor-based NPs like CdSe and ZnS QDs exert strong genotoxic effects, including DNA fragmentation and chromosomal instability, particularly in neural and muscular tissues. In contrast, AgNPs and Fe_2_O_3_ NPs trigger inflammatory responses through cytokine overexpression, notably TNF-α, IL-6, and NF-κB activation. The table also illustrates the diversity of oxidative stress biomarkers, such as MDA, p53, and caspase-3, which reflect tissue-specific toxicity pathways. Together, these findings underscore the multifactorial nature of NP-induced oxidative stress and the importance of evaluating multiple biomarkers and target organs when assessing nanotoxicological effects in zebrafish.

## 4. Experimental Approaches to Assess Oxidative Stress in Zebrafish

### 4.1. Biochemical Assays for ROS and Antioxidant Activity

Biochemical assays are fundamental for the quantification of oxidative stress markers in zebrafish (and other) tissues, providing critical insights into the impact of NPs on cellular redox balance. One of the most commonly used approaches is the measurement of lipid peroxidation through quantification of malondialdehyde (MDA) levels. MDA, an aldehydic end product of lipid peroxidation, serves as a key indicator of oxidative membrane damage. Elevated MDA levels have been consistently associated with lipid degradation and oxidative stress in NP-exposed zebrafish. For example, studies have reported increased MDA production in zebrafish embryos following exposure to AgNPs and ZnO NPs, thereby linking lipid damage to NP-induced toxicity [96].

Additionally, direct or indirect quantification of reactive oxygen species (ROS) using fluorometric or chemiluminescent methods enables the detection of oxidative stress and intracellular ROS accumulation. Among these, the DCFDA assay is widely employed for general ROS detection, while more specific fluorescent probes targeting superoxide radicals have been developed for enhanced resolution of oxidative pathways. In zebrafish models, increased ROS levels have been observed following CuO NP exposure, with maximal accumulation reported at 24 h post-exposure [97]. For example, a recent study used DHE and MitoSOX probes to trace specific ROS dynamics, providing mechanistic detail in relation to mitochondrial stress [79].

Another important biochemical indicator of oxidative imbalance is the activity of antioxidant enzymes, particularly SOD and CAT. These enzymes are commonly assessed through kinetic assays to evaluate their enzymatic activity following NP exposure. Studies have demonstrated that zebrafish exposed to TiO_2_ NPs exhibit significant reductions in both SOD and CAT activity in liver and gill tissues, suggesting depletion of antioxidant defenses and increased susceptibility to oxidative injury [88].

Recent findings also highlight that the presence of a biomolecular corona on NP surfaces can alter the outcome of these biochemical assays, either by modulating NP bioavailability or by directly affecting ROS generation and enzyme interaction profiles. This underscores the importance of considering corona-related effects when interpreting oxidative stress biomarkers in vivo.

### 4.2. Gene Expression Analysis of Oxidative Stress Pathways

Responses in molecular level which are imposed by oxidative stress-related pathways rely on the detection of changes in gene expression and provide insights into NP-induced molecular alterations. Quantitative PCR (qPCR) and RNA sequencing (RNA-seq) are commonly used practices to analyze differential gene expression in zebrafish exposed to various nanoparticles [10]. Key oxidative stress-related genes include transcription factors such as the NRF2 and KEAP1, as well as antioxidant enzymes such as SOD and GPX.

NRF2 is a central regulator of cellular defense, and its activation leads to the transcription of a wide array of antioxidant and detoxification genes. In zebrafish, increased *nrf2* expression has been observed following AgNP exposure, correlating with the upregulation of downstream antioxidant enzymes [3,98]. Similarly, *sod1*, which neutralizes superoxide radicals, is frequently upregulated in zebrafish embryos exposed to ZnO NPs, reflecting an early defensive response against ROS accumulation [34]. In contrast, exposure to CdSe quantum dots has been shown to suppress *gpx1a* expression, leading to impaired detoxification and increased apoptotic cell death in liver tissue [99].

Beyond these core markers, additional genes such as CAT, HO-1 (heme oxygenase 1), XRCC1 (DNA repair), and inflammatory markers including IL-6, TNF-α, and NF-κB have also been identified as relevant targets for assessing nanoparticle-induced stress. Comparative studies show that metal oxide NPs such as TiO_2_ induce strong upregulation of *nrf2* and *sod1*, while AgNPs provoke a broader systemic response that includes inflammatory gene activation. Polymeric and lipid-based nanoparticles tend to induce a more moderate transcriptional profile, often involving transient or limited activation of oxidative stress genes [100,101].

These gene expression signatures offer valuable insights into the mechanisms of NP toxicity, support the identification of early biomarkers, and enhance the interpretation of biochemical and phenotypic responses in zebrafish nanotoxicology studies.

### 4.3. Histopathological and Imaging Techniques

Advanced imaging techniques enable direct visualization of oxidative stress-induced damage caused by nanoparticles in zebrafish tissues. These approaches are essential for identifying the distribution of NPs and the specific sites of oxidative injury and cellular alterations at the histopathological level.

Among the most frequently used techniques is fluorescent ROS detection using DCFDA, which enables in vivo visualization of ROS accumulation in specific tissues such as the brain and liver [102]. For high-resolution analysis of subcellular alterations, TEM is employed to observe organelle damage, such as mitochondrial swelling and cristae disruption, often linked to apoptosis [103]. Standard histological staining with hematoxylin and eosin (H&E) reveals broader tissue responses, including necrosis and inflammation, particularly in organs like the gills following NP exposure [54]. Additionally, the TUNEL assay is widely used to detect DNA fragmentation and apoptotic activity in neural and hepatic tissues, serving as a marker of oxidative genotoxicity [104].

These complementary imaging strategies enhance the understanding of how nanoparticles induce oxidative stress and tissue damage, and are especially valuable in correlating morphological alterations with gene expression and biochemical biomarkers. Table 3 provides an overview of the main imaging and histopathological techniques applied in zebrafish to assess oxidative damage, along with their specific findings and applications.

### 4.4. Behavioral and Physiological Endpoints

Oxidative stress induced by NPs can result in a range of behavioral and physiological abnormalities in zebrafish, serving as sensitive, non-invasive biomarkers of neurotoxicity and systemic dysfunction. Zebrafish models are particularly well suited for behavioral analysis due to their transparent larvae, rapid development, and conserved neurological pathways.

Alterations in swimming activity are commonly reported following nanoparticle exposure. Reduced motility, hypoactivity, and erratic swimming patterns are frequently observed and are often linked to oxidative damage in neural tissue. For instance, AgNP exposure has been shown to decrease spontaneous locomotion and elicit anxiety-like behavior in zebrafish larvae, correlating with elevated ROS levels in the brain [111,112]. Similarly, cardiotoxicity induced by CuO NPs has been documented through alterations in heart rate, including bradycardia and tachycardia, effects attributed to mitochondrial dysfunction and ROS accumulation in cardiac tissues [80,113].

Developmental malformations also represent a critical endpoint associated with oxidative stress, including craniofacial deformities, pericardial edema, and spinal curvature. TiO_2_ NP exposure in zebrafish embryos has been shown to significantly increase these malformations, primarily due to ROS-mediated DNA damage during early development [10,87]. Additionally, respiratory dysfunction has been observed in zebrafish exposed to Fe_2_O_3_ NPs, with rapid opercular movement indicating respiratory distress and histopathological analysis revealing gill damage and NF-κB-driven inflammatory responses [10,29].

Beyond general motor activity, stimulus-evoked behavioral responses offer refined insight into NP-induced neurotoxicity. The acoustic startle response and visual motor response are widely applied to assess sensorimotor reflexes in zebrafish larvae. These tests evaluate changes in movement in response to sound or light stimuli and have been validated as sensitive indicators of nervous system impairment. For example, exposure to ZnO NPs and AgNPs has been associated with attenuated startle reflexes and abnormal responses to light–dark transitions, suggesting disruption of central and peripheral neural pathways [114,115]. Studies by Tal et al. demonstrated that behavioral profiling, including startle and photomotor tests, can predict nanomaterial toxicity even at sub-lethal doses, making them valuable for early hazard screening [116]. Similarly, Dickmeiss et al. developed automated startle response tools to quantify neurodevelopmental toxicity of various engineered nanoparticles with high sensitivity [117,118].

Overall, these physiological and behavioral markers provide a holistic view of nanoparticle toxicity in zebrafish and offer valuable translational insight into potential human health risks.

## 5. Pharmaceutical Applications of Nanoparticles Using Zebrafish (*Danio rerio*) Models

In the context of modern toxicology and nanomedicine development, zebrafish embryos have emerged as a valuable tool aligned with the principles of the 3Rs strategy (Replacement, Reduction, and Refinement). Their legal status as non-protected animals prior to the onset of independent feeding, combined with their genetic and physiological similarities to higher vertebrates, make them ideal for high-throughput testing with reduced ethical burden [119]. As part of New Approach Methodologies (NAMs), zebrafish models are increasingly adopted in pharmacological and environmental safety assessments, offering predictive insights while minimizing reliance on traditional mammalian models [120,121]. Additionally, zebrafish contribute to the growing field of phylotoxicology, which emphasizes the conservation of toxicity mechanisms across evolutionary lineages. Supported by European consortia such as PrecisionTox, this approach leverages evolutionary relationships to improve risk assessment frameworks and enhance the relevance of non-mammalian models [122]. Building on these principles, zebrafish models offer unique advantages for the evaluation of nanoparticle-based therapeutics, enabling efficient in vivo screening while adhering to ethical and scientific innovation frameworks [123,124].

Zebrafish are an essential vertebrate model in nanomedicine research due to their high genetic homology to humans and feasibility to employ in the laboratory setting for large-scale experiments. Their high fecundity enables large-scale pharmacological screenings, and their transparent embryos allow real-time tracking of NP biodistribution and interactions in vivo. Zebrafish have been successfully used for toxicity screening, drug efficacy evaluations, and nanoparticle-mediated therapeutic interventions [114]. Studies indicate that selenium nanoparticles (Se NPs), quercetin-functionalized AuNPs, and curcumin-loaded NPs significantly enhance antioxidant enzyme activity in zebrafish tissues, mitigating ROS-related toxicity [115,125,126]. Recent findings highlight the potential of nanoencapsulated polyphenols in promoting cellular defense mechanisms against oxidative stress [123]. AuNPs associated with curcumin have been shown to exert neuroprotective effects in zebrafish models of oxidative stress, reducing neuronal apoptosis by 40% and significantly upregulating antioxidant enzymes (SOD, GPx, and CAT) in brain tissues [127]. Another study demonstrated that curcumin-loaded polymeric nanoparticles increased zebrafish survival rates in oxidative stress models by 50% compared to free curcumin administration [128]. Additionally, lipid-based antioxidant NPs loaded with resveratrol demonstrated enhanced bioavailability and anti-inflammatory effects in zebrafish models of cardiovascular disease, reducing oxidative stress markers and preventing endothelial dysfunction [129,130].

### 5.1. Nanoparticles as Antioxidant Therapeutics in Pharmaceutical Sciences

Nanoparticles infused with antioxidants show significant potential in the treatment of oxidative stress-related diseases, including neurodegenerative disorders, cardiovascular conditions, and cancer. By acting as ROS scavengers, these NPs can mitigate oxidative damage, prevent inflammatory cascades, and improve cellular resilience. Numerous studies indicate that NP-based antioxidant systems efficiently neutralize ROS, thereby protecting cells from oxidative damage [8]. For example, cerium oxide (CeO_2_) NPs exhibit intrinsic catalytic activity that mimics SOD and CAT, making them highly effective in oxidative stress models [131]. A study on zebrafish oxidative stress models found that CeO_2_ NPs reduced lipid peroxidation by 45% and significantly improved neuronal survival rates. The catalytic nature of CeO_2_ NPs helped restore normal antioxidant enzyme function, effectively reducing neurotoxicity in zebrafish larvae exposed to oxidative stress-inducing compounds [132]. Other research has demonstrated that selenium nanoparticles (SeNPs) and AuNPs functionalized with flavonoids provide neuroprotection in zebrafish models of Parkinson’s disease and Alzheimer’s disease by preventing oxidative damage and modulating inflammatory pathways [133,134].

### 5.2. Natural Antioxidants Used in NP Formulations

The incorporation of natural antioxidants into NP formulations enhances their therapeutic potential while reducing toxicity. This information is detailed in Table 4.

### 5.3. Drug Delivery and Biocompatibility Testing

Advanced imaging techniques, such as fluorescence imaging, confocal microscopy, and bioluminescence tracking, allow for real-time visualization of NP distribution in zebrafish models. Studies using quantum dots (QDs) and radiolabeled NPs have demonstrated their ability to monitor NP uptake, accumulation, and clearance in different organs, offering valuable pharmacokinetic insights [140].

Zebrafish models have been extensively used to analyze NP absorption, circulation, metabolism, and excretion. Studies on AuNPs and polymeric nanocarriers have shown their prolonged circulation times and biodistribution in zebrafish embryos, mimicking pharmacokinetic profiles observed in mammalian models [141]. Research on lipid-based NPs has also indicated improved bioavailability, making them suitable carriers for hydrophobic drugs [142,143].

Polylactic-co-glycolic acid (PLGA) NPs loaded with antioxidants have exhibited controlled drug release in zebrafish models, demonstrating sustained therapeutic effects, enhanced bioavailability, and reduced systemic toxicity. A study on curcumin-loaded PLGA NPs reported prolonged circulation and targeted accumulation in zebrafish liver, leading to a 50% reduction in ROS levels compared to free curcumin administration. Furthermore, chitosan-coated PLGA nanoparticles encapsulating vitamin E exhibited sustained release and antioxidant protection, significantly improving zebrafish survival rates under oxidative stress conditions and restoring mitochondrial integrity [128,144,145].

### 5.4. Balancing Toxicity Versus Therapeutic Potential

Zebrafish-based oxidative stress models allow researchers to assess how different nanoparticle compositions and dosages affect biological systems and provide insights into their long-term safety profiles.

Studies have shown that determining the safe concentration range for therapeutic nanoparticles (NPs) is crucial to minimize off-target effects. Research involving AuNPs and polymeric nanocarriers in zebrafish models has demonstrated that precise dosing can reduce cytotoxicity while enhancing drug delivery efficiency. For instance, a study on the toxicity of various nanomedicine materials, including gold and iron oxide nanoparticles, evaluated their effects in zebrafish embryos, highlighting the importance of assessing safe concentration ranges to minimize adverse effects [54]. Moreover, during our own experience with silver NPs we observed a striking contrast in the biological effects of plant extracts when administered alone versus when incorporated into silver nanoparticle formulations. When used independently, the extracts demonstrated significant antioxidant and neuroprotective effects without any detectable toxicity. However, upon integration within silver NPs, at the same dose and under identical experimental conditions, we noticed an unexpected increase in toxicity. These findings highlight the complexity of NP-mediated substance delivery, where interactions between the nanocarrier and the bioactive compound can alter pharmacodynamics and toxicity. Based on such discrepancies, comprehensive pharmacokinetics studies are essential in order to elucidate how NPs formulations can influence absorption, distribution and metabolism of therapeutic compounds. Understanding these mechanisms is imperative in order to properly balance the therapeutic potential of NPs formulations while mitigating possible unexpected and unintended toxicological effects.

Other studies on chronic NP exposure in zebrafish revealed potential risks associated with prolonged usage. AgNPs and TiO_2_ NPs have been found to accumulate in the liver and the brain of zebrafish, leading to oxidative damage and inflammatory responses over extended periods [24,146]. Research on lipid-based NPs suggests that their biodegradable nature may reduce long-term toxicity risks, making them promising candidates for pharmaceutical applications [147].

Furthermore, a study on iron oxide (Fe_3_O_4_) NPs demonstrated that chronic exposure in zebrafish induced significant oxidative stress and neurotoxicity, highlighting the need for careful evaluation of NP formulations before their use in clinical settings [148].

## 6. Conclusions

The findings of this review highlight the significant role of oxidative stress in NP-induced toxicity with emphasis on the zebrafish model. Studies have demonstrated that various nanoparticles, including metal-based (AgNPs, TiO_2_ NPs, Fe_2_O_3_ NPs), polymeric, lipid-based, and quantum dots, can induce oxidative stress through ROS overproduction, mitochondrial dysfunction, inflammation, and genotoxicity. These toxicological effects are strongly influenced by the physicochemical characteristics of nanoparticles, including size, shape, surface charge, and core composition. Recent insights also highlight the role of protein corona formation, which can significantly alter nanoparticle behavior by modifying surface properties, bioavailability, and cellular uptake. Such parameters must be carefully considered when evaluating the oxidative potential and biological interactions of nanoparticle systems. Zebrafish have emerged as a versatile and reliable model organism for evaluating NP-induced oxidative stress, offering advantages such as genetic similarity to humans, rapid development, and transparent embryos that allow real-time assessment of oxidative damage and pharmacological responses.

From a pharmaceutical perspective, NPs functionalized with antioxidants, such as curcumin, resveratrol, quercetin, and selenium nanoparticles, have shown promising therapeutic applications in neuroprotection, hepatoprotection, and cardiovascular disease management. Zebrafish models have been instrumental in screening NP formulations, assessing biodistribution, and optimizing drug delivery systems to balance efficacy with safety. Toxicological studies using this model have provided valuable insights into the long-term risks associated with NP accumulation, guiding the development of safer nanopharmaceuticals. Moreover, the use of zebrafish embryos aligns well with the ethical principles of the 3Rs strategy and is widely embraced within the framework of NAMs for modern toxicology. Their increasing adoption enables reliable, high-throughput screening of nanoparticle safety and therapeutic performance, while reducing dependence on traditional mammalian models. In addition, this model organism contributes to the emerging field of phylotoxicology, which investigates evolutionarily conserved toxicity pathways and offers translational insights relevant to both human and environmental health. Despite these advances, standardized methodologies for oxidative stress assessment in zebrafish models remain a critical need. Future research should focus on long-term NP exposure studies, multi-generational toxicity assessments, and the integration of omics technologies (transcriptomics, proteomics, and metabolomics) to deepen the understanding of NP interactions with biological systems. A comprehensive evaluation of NP-induced oxidative stress should integrate molecular markers (e.g., differential gene expression of nrf2, sod1, gpx1a), advanced imaging and histopathological techniques (such as DCFDA fluorescence, TEM, H&E staining, and TUNEL assays), as well as behavioral endpoints including locomotor activity, heart rate variability, and stimulus-evoked responses like the acoustic startle test. These complementary approaches increase mechanistic understanding and enhance the predictive power of zebrafish models in both pharmaceutical development and environmental nanotoxicology.

Furthermore, advancing the biodegradability and biocompatibility of NP formulations will be essential in mitigating potential toxic effects while maximizing therapeutic benefits. In conclusion, zebrafish models provide an effective and high-throughput platform for evaluating both the toxicity and therapeutic potential of nanoparticles. Understanding NP-induced oxidative stress mechanisms will not only facilitate the design of safer nanomaterials but also accelerate the development of novel antioxidant-based nanomedicines for various biomedical applications.

Future studies should consider expanding multi-generational studies (long-term exposure studies on zebrafish should be conducted to assess how NP-induced oxidative stress affects successive generations, particularly in the context of epigenetic modifications and inherited toxicity), personalized nanomedicine (develop patient-specific nanotherapeutics, particularly for neurodegenerative diseases, cancer, and metabolic disorders), and green nanotechnology approaches (develop environmentally friendly and biocompatible NPs using sustainable materials to reduce toxicity risks while maintaining therapeutic efficacy).

## Figures and Tables

**Table 1:** Impact of NP size on oxidative stress and toxicity.

Nanoparticle Type	Small Size (≤20 nm)	Medium Size (20–50 nm)	Large Size (>50 nm)	Reference
Ag NPs	Mitochondrial Dysfunction	Moderate ROS	Minimal ROS	[11,19,22,25]
Au NPs	Pro-inflammatory Effects	Minimal Effects	Low ROS	[19,26,27]
Cu NPs	DNA Damage	Inflammation	Low ROS	[28]
Fe NPs	Cellular Damage	Mitochondrial Dysfunction	Low ROS	[29,30]
ZnO NPs	Apoptosis	Moderate ROS	Low ROS	[31,32,33,34]
TiO_2_ NPs	Neurotoxicity, Behavioral Disruptions	Moderate ROS	Minimal ROS	[23,24]
CuO NPs	Genotoxicity	Cellular Stress	Minimal ROS	[35,36,37]
Fe_2_O_3_ NPs	DNA Fragmentation	Cellular Inflammation	Low ROS	[36,38,39,40,41]
CdSe QDs	DNA Damage, Apoptosis	Mitochondrial Dysfunction	Low ROS	[42,43]
ZnS QDs	Phototoxicity, Lipid Peroxidation	DNA Damage	Limited Cytotoxicity	[42,43]

**Table 2:** Effects of nanoparticles on oxidative stress mechanisms in zebrafish.

Nanoparticle Type	Affected Organ/System	Mechanism of Toxicity	Key Biomarkers/Cytokines	Observed Effects	Case Study	Reference
TiO_2_ NPs	Liver, Gills	ROS Overproduction, Antioxidant Enzyme Suppression	↓ SOD, ↓ CAT, ↑ MDA	Oxidative stress-mediated toxicity	Enzyme activity decreased in liver	[24,48]
CuO NPs	Brain	Mitochondrial Dysfunction, Apoptotic Pathways	↑ p53, ↑ Caspase-3, ↓ ATP	ROS-induced neurotoxicity	Mitochondrial apoptosis triggered	[48,80,82]
Fe_2_O_3_ NPs	Gills, Immune System	NF-κB Activation, Inflammatory Response	↑ IL-8, ↑ IL-1β, ↑ NF-κB	Respiratory dysfunction, chronic inflammation	NF-κB-mediated gill inflammation	[29,83]
AgNPs	Liver	Hepatotoxicity, Cytokine Overexpression	↑ TNF-α, ↑ IL-6	Liver inflammation, oxidative damage	TNF-α and IL-6 upregulation	[29,83,84]
ZnO NPs	Muscle, Liver	DNA Damage, Lipid Peroxidation	↑ DNA Strand Breaks, ↑ MDA	Chromosomal instability, apoptosis	DNA strand breaks observed	[8,34]
CdSe QDs	Brain, Muscle	Genotoxicity, Lipid Peroxidation	↑ XRCC1, ↑ p53	DNA fragmentation, neuronal dysfunction	Genotoxicity in larvae	[9,26,85]

**Table 3:** Imaging and Histopathological Techniques for Oxidative Stress Assessment.

Technique	Purpose	Application in Zebrafish	Key Findings	Reference
Fluorescent ROS Detection (DCFDA Assay)	Measures intracellular ROS levels	Used to quantify oxidative stress in zebrafish tissues	AgNP-exposed zebrafish embryos show significant ROS increase in brain and liver	[94,105,106]
Transmission Electron Microscopy (TEM)	Ultrastructural analysis of NP localization and organelle damage	Identifies mitochondrial swelling, cristae disruption, and vacuolization	TiO_2_ NPs accumulate in hepatic mitochondria, leading to apoptosis	[76,107]
Hematoxylin and Eosin (H&E) Staining	Histopathological assessment of tissue damage and inflammation	Detects necrosis, epithelial degeneration, and immune cell infiltration	CuO NP exposure causes epithelial damage and chronic inflammation in zebrafish gills	[108]
TUNEL Assay (Terminal deoxynucleotidyl transferase dUTP Nick End Labeling)	Identifies apoptotic DNA fragmentation	Used to assess neurotoxicity and genotoxicity in zebrafish tissues	CdSe QD exposure leads to increased TUNEL-positive cells in zebrafish brain	[109,110]

**Table 4:** Natural antioxidants in nanoparticle formulations for oxidative stress reduction.

Type of Antioxidant- Functionalized NP	Mechanism of Action	Effect in Zebrafish Models	Reference
Polyphenol-coated NPs (quercetin, catechins)	Enhance oxidative stress resistance by scavenging ROS	Reduce ROS levels and increase antioxidant enzyme activity in zebrafish tissues	[57,135,136]
Resveratrol-loaded liposomes	Improve bioavailability and stability of antioxidant compounds	Prevent lipid peroxidation and DNA damage in oxidative stress-exposed zebrafish embryos	[130,137]
Plant-derived antioxidant NPs	Provide biocompatible approaches for reducing oxidative toxicity	Decrease apoptosis and inflammatory response in zebrafish liver and brain tissues	[138,139]
Liposomal curcumin NPs	Target oxidative damage by decreasing ROS accumulation and restoring mitochondrial function	Reduce oxidative damage in zebrafish liver tissue and improve metabolic activity	[128,129]

## Data Availability

No new data were created.

## Abbreviations

The following abbreviations are used in this manuscript:

NPs	Nanoparticles
ROS	Reactive oxygen species
CAT	Catalase
SOD	Superoxide dismutase
GPx	Glutathione peroxidase
DNA	Deoxyribonucleic acid
MDA	Malondialdehyde
DCFDA	2′,7′-dichlorofluorescin diacetate
RNA	Ribonucleic acid
ATP	Adenosine triphosphate
IL	Interleukin
TNF-α	Tumor necrosis factor alpha
PCR	Polymerase chain reaction
KEAP	Kelch-like ECH-associated protein 1
NRF2	Nuclear factor erythroid 2
